# The *Pseudoalteromonas luteoviolacea* L-amino Acid Oxidase with Antimicrobial Activity Is a Flavoenzyme

**DOI:** 10.3390/md16120499

**Published:** 2018-12-12

**Authors:** Andrés Andreo-Vidal, Antonio Sanchez-Amat, Jonatan C. Campillo-Brocal

**Affiliations:** 1 Department of Genetics and Microbiology, University of Murcia, 30100 Murcia, Spain; andres.andreo@um.es; 2 Department of Industrial Biotechnology, School of Engineering Sciences in Chemistry, Biotechnology and Health (CBH), KTH Royal Institute of Technology, AlbaNova University Center, SE-106 91 Stockholm, Sweden

**Keywords:** L-amino acid oxidase, antimicrobial activity, flavin cofactor, *Pseudoalteromonas luteoviolacea*

## Abstract

The marine environment is a rich source of antimicrobial compounds with promising pharmaceutical and biotechnological applications. The *Pseudoalteromonas* genus harbors one of the highest proportions of bacterial species producing antimicrobial molecules. For decades, the presence of proteins with L-amino acid oxidase (LAAO) and antimicrobial activity in *Pseudoalteromonas luteoviolacea* has been known. Here, we present for the first time the identification, cloning, characterization and phylogenetic analysis of Pl-LAAO, the enzyme responsible for both LAAO and antimicrobial activity in *P. luteoviolacea* strain CPMOR-2. Pl-LAAO is a flavoprotein of a broad substrate range, in which the hydrogen peroxide generated in the LAAO reaction is responsible for the antimicrobial activity. So far, no protein with a sequence similarity to Pl-LAAO has been cloned or characterized, with this being the first report on a flavin adenine dinucleotide (FAD)-containing LAAO with antimicrobial activity from a marine microorganism. Our results revealed that 20.4% of the sequenced *Pseudoalteromonas* strains (specifically, 66.6% of *P. luteoviolacea* strains) contain *Pl-laao* similar genes, which constitutes a well-defined phylogenetic group. In summary, this work provides insights into the biological significance of antimicrobial LAAOs in the *Pseudoalteromonas* genus and shows an effective approach for the detection of novel LAAOs, whose study may be useful for biotechnological applications.

## 1. Introduction

Marine ecological niches are excellent sources for many bioactive compounds of biotechnological and pharmaceutical interest. Among them, antimicrobial compounds synthetized by marine organisms offer a promising alternative to antibiotics [1]. Microorganisms from the *Pseudoalteromonas* genus are well-known producers of several metabolites with antimicrobial activity, such as alkaloids, polyketides, peptides, and proteins [2,3]. The genus *Pseudoalteromonas* includes Gram-negative, heterotrophic, and aerobic marine bacteria, which belong to the *Alteromonadales* order in the *Gammaproteobacteria* class. They are commonly found in association with seawater macroorganisms, where they play a critical role in holobiont homeostasis through their metabolic activities [2]. *Pseudoalteromonas luteoviolacea* was described in 1976, and was reported to produce antibacterial polyanionic substances inhibited by catalase.

**Figure 1:**
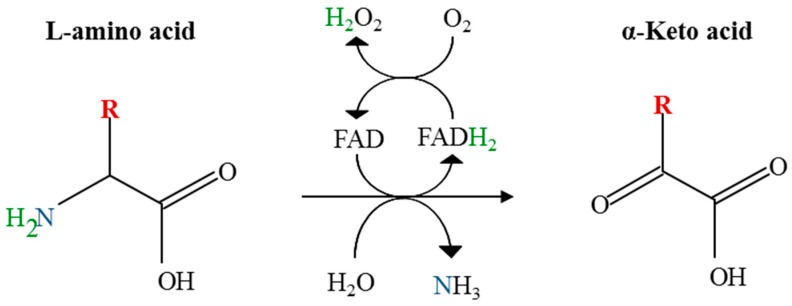
Reaction catalyzed by flavin adenine dinucleotide (FAD)-dependent L-amino acid oxidases (LAAOs).

L-Amino acid oxidases (LAAOs) are enzymes that catalyze the oxidative deamination of amino acids, releasing the corresponding keto acid, ammonium, and hydrogen peroxide. Two groups of these enzymes have been described so far. The best-known group (EC 1.4.3.2) utilizes flavin adenine dinucleotide (FAD) as a cofactor, and oxidizes amino acids in the alpha position (Figure 1). The second group has recently been described as containing a quinone cofactor generated by post-translational modification of the protein [4]. This group possesses a distinct evolutionary origin [5] and has been named as LodA-like proteins, after the description of LodA, a L-lysine epsilon-oxidase [6]. LAAOs are distributed in many biological groups including bacteria, fungi, algae, plants, insects, molluscs, fishes, and mammals (including humans), although the most studied members of this group are the LAAOs present in snake venom [7,8]. The generation of hydrogen peroxide gives to these enzymes antimicrobial properties that have been related to different physiological processes, such as the biocontrol agents in fungi against microbial competitors [9] or protection of fish skin and gills from bacterial infections [10]. LAAOs are of great biotechnological interest in different applications, such as the design of biosensors, biotransformations, or biomedicine, and are attracting more and more interest because of their broad and relevant biological functions [7,11].

LAAOs from marine bacteria are believed to play important biological and ecological roles in their niches, mediated by antimicrobial activity. This is the case of the L-lysine ε-oxidases from the gammaproteobacteria *Marinomonas mediterranea* (LodA) and *Pseudoalteromonas tunicata* (AlpP), which are involved in biofilm development and dispersal through hydrogen peroxide generation [1,12]. Some other LAAOs with an antimicrobial activity have been described and characterized in different marine bacteria. PfaP from *Pseudoalteromonas flavipulchra* JG1 shares a high similarity with AlpP and LodA [13]. *P. flavipulchra* C2 was reported to have a LAAO of a broad substrate range, oxidizing L-Lys, L-Met, L-Glu, L-Leu, L-Gln, L-Tyr and L-Phe, which contained a nine amino-acid fragment similar to AlpP [14]. *P. luteoviolacea* produces a protein with an L-amino acid oxidase (LAAO) activity of a broad-substrate spectrum, in which the hydrogen peroxide generated mediates its antimicrobial activity, although the gene coding for this enzyme has not been identified [15,16]. *Aquimarina* sp. antisso-27 also synthesizes a broad spectrum LAAO (L-Leu > L-Ile > L-Met > L-Val), with both antibacterial and algicidal activity [17]. It is worth mentioning that all of these antimicrobial LAAOs are synthesized by bacteria associated with the microbiota of higher marine organisms, and none of them have been described with a flavin cofactor [5].

Recently, our group has reported the presence of PlGoxA, a LAAO very specific for Gly synthetized by *P. luteoviolacea* CPMOR-2 [18]. PlGoxA contains a cysteine tryptophylquinone (CTQ) cofactor generated by the post-translational modification of two residues in the same protein. This protein bears similarity to GoxA from *M. mediterranea* [19]. Regardless, PlGoxA did not show a broad substrate range, and thus the LAAO with an antimicrobial activity described in *P. luteoviolacea* CPMOR-2 still remains to be determined [16]. In the present study, we show that the antimicrobial activity of *Pseudoalteromonas luteoviolacea* is due to an L-amino acid oxidase with a flavin cofactor. We believe that our findings provide insights into the biological and ecological significance of antimicrobial LAAOs in the *Pseudoalteromonas* genus.

## 2. Results

### 2.1. Identification of the Gene Encoding the LAAO Activity in CPMOR-2 Strain

As reported by Gómez et al. (2008), *Pseudoalteromonas luteoviolacea* CPMOR-2 synthesizes a protein with an antimicrobial and amino acid oxidase activity, although the gene encoding this protein had not been identified. With this purpose, first, we investigated the optimal conditions for the expression of LAAO activity. Hence, *P. luteoviolacea* CPMOR-2 was inoculated in different conditions and the synthesis of L-amino acid oxidases was evaluated using 2% casamino acids as a substrate. The maximal production was observed in the supernatant at the stationary phase of growth in medium MNGY (Supplementary Figure S1).

**Figure 2:**
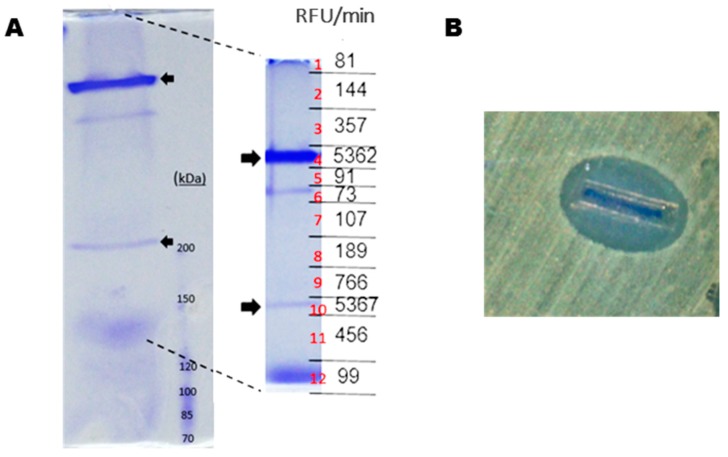
Identification of proteins with LAAO and antimicrobial activity in *P. luteoviolacea* CPMOR-2. (**A**) SDS-PAGE of concentrated supernatants of CPMOR-2 strain grown in MNGY medium (See materials and methods). Arrows point to the protein bands with LAAO activity measured against casamino acids. LAAO activity for each gel fragment is expressed as relative fluorescence units per min (RFU/min). (**B**) Antibiograms against *E. coli* UM202 of fragment 4 sliced from gel in A showing antimicrobial activity.

Next, *P. luteoviolacea* CPMOR-2 was cultivated until stationary phase in MNGY, and the supernatant was collected and concentrated using centrifugal filters of a 30 kDa cut-off. These samples were run under SDS-PAGE in non-denaturing conditions for LAAO activity (see material and methods), and slices were cut from the gel and evaluated for LAAO and antimicrobial activities. Two protein bands (fragments 4 and 10) were detected with LAAO activity (Figure 2A). In addition, from a set of parallel identical lines, those two bands with LAAO activity were excised, and the trypsin was digested, subjected to HPLC-MS/MS and analyzed against *P. luteoviolacea* CPMOR-2 genome. It was observed that various peptides from the upper band matched the protein with GenBank accession number KZN49687 (with a coverage of 22%). This protein has been named in this work as Pl-LAAO. The second band with low molecular mass corresponded to PlGoxA (accession WP_063358237) (coverage of 16%), a LAAO with quinone cofactor specific for glycine, which was recently described [18]. The upper band showed a strong antimicrobial activity (Figure 2B). Accordingly, we hypothesized that the upper band may correspond to the LAAO with a broad substrate range previously reported in *P. luteoviolacea* CPMOR-2 [16].

### 2.2. Sequence Analysis of Pl-LAAO

Pl-LAAO contains 653 amino acids, with a predicted molecular weight of 74 kDa and an isoelectric point of 4.85 (http://web.expasy.org/compute_pi/). In spite of being detected in the supernatants of the cultures, Pl-LAAO does not contain any classical or non-classical signal peptide according to the predictor web tools SignalP 4.1 (http://www.cbs.dtu.dk/services/SignalP/) and SecretomeP 2.0 (http://www.cbs.dtu.dk/services/SecretomeP). Other web servers, like PSORTb (http://www.psort.org/psortb/) or CELLO (http://cello.life.nctu.edu.tw/), were not able to provide an accurate prediction of the subcellular location of Pl-LAAO.

**Figure 3:**
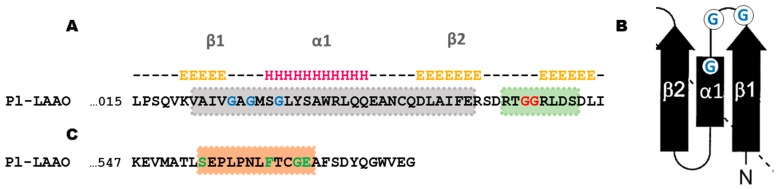
Conserved flavin-binding motifs in Pl-LAAO peptide sequence from *P. luteoviolacea* CPMOR-2. (**A**) The dinucleotide binding motif (DBM) domain (highlighted in grey) and GG-motif (highlighted in green) in the N-terminal region of the Pl-LAAO sequence. The three conserved Gly in the DBM domain are in blue, while the two conserved Gly in the GG-motif are in red. The secondary structure was predicted using the tool “JPred 4” (http://www.compbio.dundee.ac.uk/jpred/). H: alpha helix; E: beta sheet; -: disordered structure. (**B**) First half of the classic Rossmann fold topology. The arrows designate β-strands and rectangles denote α-helices. (**C**) Conserved motif in the C-terminal region (highlighted in red) of proteins from the glutathione reductase family. Conserved residues are in green.

The Pl-LAAO sequence presents a similarity from positions 28–440 to the pfam01593 domain, which is characteristic of amino oxidases. The sequence analysis also showed in Pl-LAAO some conserved motifs of the FAD/NAD(P)-binding domain superfamily [20]. For instance, the conserved dinucleotide binding motif (DBM), with the consensus sequence xhxhGxGxxGxxxhxxh(x)8hxhE(D) (where x is any residue and h is a hydrophobic residue) is located in the N-terminus (Figure 3A). In addition, when analyzing the prediction of the secondary structure of this N-terminal region with JPred4 (http://www.compbio.dundee.ac.uk/jpred/), a structure β1α1β2 is clearly observed, in which the arrangement of the glycines coincides with the characteristic glycine-rich phosphate-binding loop of the Rossmann fold [21] (Figure 3B). Shortly after the DBM domain in the Pl-LAAO sequence, it is possible to recognize the “GG motif” (RxGGRxxS/T), which is a common feature in LAAOs sequences [22]. Another conserved FAD-binding sequence motif is an eleven amino acid segment, T(S)xxxxxF(Y)xxGD(E), which has been described in the C-terminal region of proteins from the glutathione reductase family [20]. The only difference is that this motif in the Pl-LAAO sequence shows an additional residue between S and F (Figure 3C).

In terms of sequence, as far as we know, no similar protein to Pl-LAAO has been described so far. For instance, the only FAD-LAAO from a *Pseudolteromonas* specie reported hitherto, which presents a homology with L-aspartate oxidases, shares only a 14.3% identity with Pl-LAAO [23]. Achacin, from the giant African snail *Achatina fulica*, was found to be the most similar protein to Pl-LAAO that has been already characterized, with a 20.4% identity [24].

### 2.3. Recombinant Expression and Partial Biochemical Characterization of Pl-LAAO

**Figure 4:**
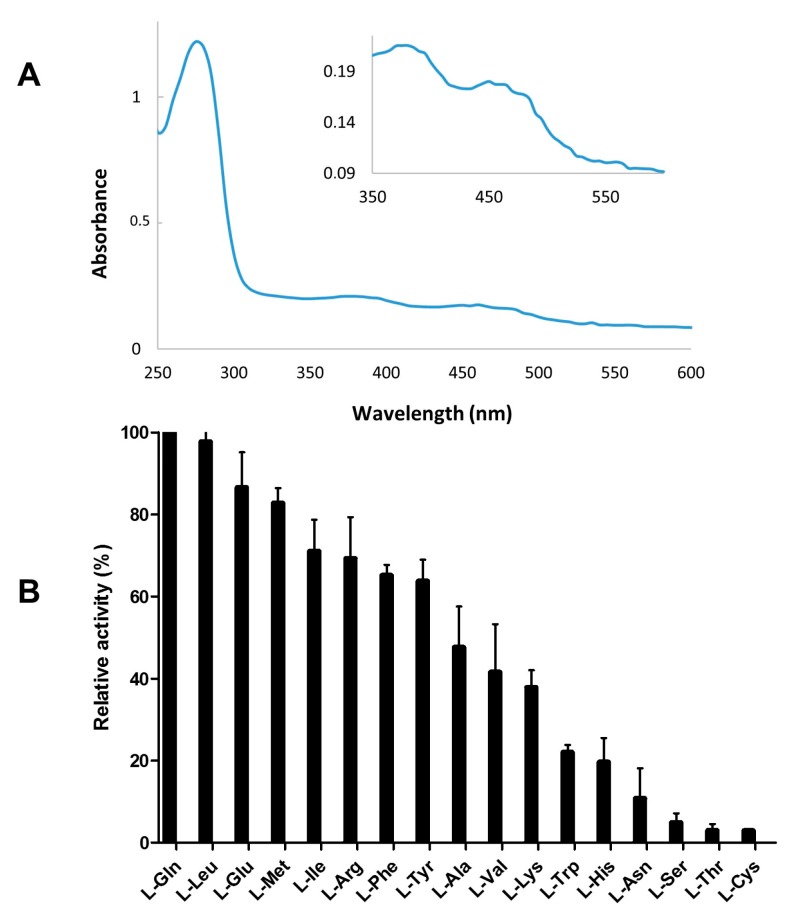
(**A**) UV-VIS spectrum of purified recombinant Pl-LAAO enzyme solution. (**B**) LAAO activity spectrum of Pl-LAAO. Values are expressed as percentage of the activity on the best substrate. The 20-protein standard amino acids were assayed at 2 mM, but only the oxidized ones are shown.

To further confirm that *Pl-laao* encodes the protein with LAAO and the antimicrobial activity observed in the supernatant of strain CPMOR-2, this gene was cloned into pET15b fused to a poly-His tag. It was recombinantly expressed in *E. coli* CD03, which is a strain mutated in a catalase, allowing for the detection of enzymatic activities releasing hydrogen peroxide in cell extracts [25]. After purification by a Ni-NTA agarose column and a buffer exchange to remove imidazole, the samples were found to be yellow, probably indicating the presence of a flavin cofactor. A UV-VIS spectroscopy analysis showed the characteristic absorption spectrum between 300 and 500 nm that is typical for flavoproteins [26], with absorption peaks at 370 and 460 nm (Figure 4A). The recombinant purified Pl-LAAO sample was subjected to LAAO activity assays with the 20-standard protein α-L-amino acids in order to determine the substrate spectrum. It was observed that Pl-LAAO shows a broad substrate range, with L-Gln and L-Leu being the preferred substrates. Pl-LAAO also oxidizes L-Glu > L-Met > L-Ile > L-Arg > L-Phe > L-Tyr > L-Ala > L-Val > L-Lys > L-Trp > L-His > L-Asn > L-Ser > L-Thr and L-Cys (Figure 4B).

**Figure 5:**
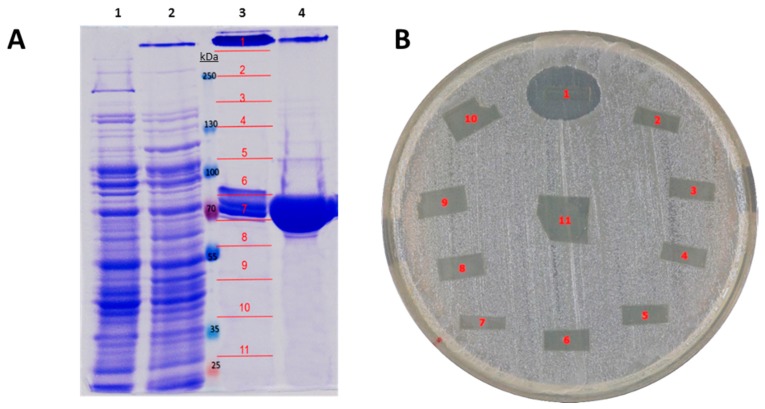
Detection in non-denaturing SDS-PAGE of recombinant Pl-LAAO activity. (**A**) SDS-PAGE of crude extract and purified Pl-LAAO samples. (1) Crude extract of *E. coli* CD03 containing pET-15b with no insert. (2) Crude extract of *E. coli* CD03 containing pET-15b with *Pl-laao* as an insert. (3) Purified Pl-LAAO sample with no treatment. (4) Purified Pl-LAAO sample boiled at 95 °C for 5 min. (**B**) A parallel lane similar to lane 3 was sliced to perform antibiograms against *E. coli* UM202 in a Luria-Bertani (LB) medium.

A purified sample of the recombinant Pl-LAAO was run in SDS-PAGE. Two bands were observed, one of them showed the expected molecular mass of the monomer conformation (74 kDa). The second band with a high molecular mass may correspond to a Pl-LAAO multimeric complex, probably a homotetramer, according to its apparent molecular mass. The gel was sliced in different fragments for antimicrobial and enzymatic activity measurements. The results revealed that only the multimeric form (slice number 1) exhibited the LAAO and antimicrobial activity (Figure 5). The monomeric form did not present either antimicrobial or LAAO activity. This result indicates that the active conformation of the enzyme is a multimeric complex, which is in agreement with the size of the active proteins in the gels of the supernatants of *P. luteoviolacea* (Figure 2).

**Figure 6:**
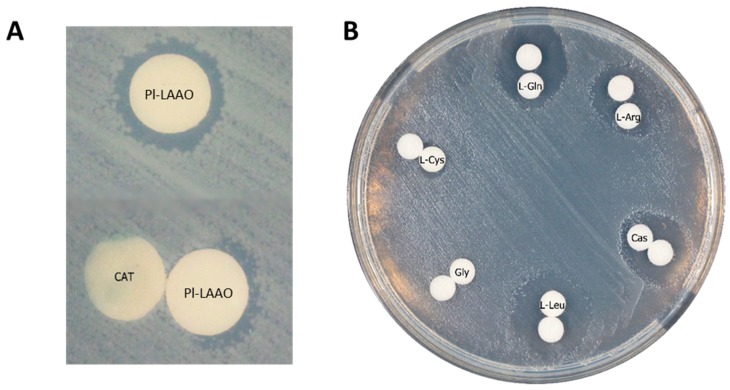
Antibiograms of purified recombinant Pl-LAAO against *E. coli* UM202. (**A**) Antibiograms in LB. Pl-LAAO disk loaded with 20 μL of purified protein at 7 × 10^−2^ mg mL^−1^. CAT—disk loaded with 20 μL of catalase 10 mg mL^−1^. (**B**) Antibiogram in M9 medium in the presence of some amino acids at 50 mM as a substrate. White disks contained 20 μl of Pl-LAAO at 0.25 mg mL^−1^. Cas—casamino acids 10%.

To confirm that the antimicrobial activity was due to the hydrogen peroxide generated by the LAAO activity, distinct experiments were performed. First, it was observed that the antibacterial effect of Pl-LAAO was inhibited by catalase in the antibiograms against *E. coli* UM202 in a Luria Bertani (LB) medium (Figure 6A). In the chemically defined medium M9, no antimicrobial activity was detected unless amino acids were supplemented. It was observed that the antimicrobial activity correlated with the hydrogen peroxide production in the oxidation of the amino acid by the LAAO. Thus, L-Gln and L-Leu, which are the preferred substrates of Pl-LAAO, produced the bigger halos, whereas the Gly addition did not allowed for the detection of the inhibition halo, as it is not a substrate for the enzyme (Figure 6B). These results suggest that hydrogen peroxide mainly responsible for the antimicrobial activity, although it cannot be completely ruled out that some intermediates may potentiate its effect, similarly to the case of some lysine alpha-oxidases [27].

### 2.4. Detection, Distribution, and Phylogenetic Analysis of Proteins Similar to Pl-LAAO

In order to identify similar proteins to Pl-LAAO in the microbial genomes, we did a BLASTp search against the Integrated Microbial Genomes (IMG) database of the genome sequences as of 14 September 2018. Using the Pl-LAAO peptide sequence as a query and a cut-off limit for the E-value of 1e^−10^, we found 57 genes encoding similar proteins to Pl-LAAO. A total of eight genes were not included in the final analysis. Six of them were repetitive genes with different accession numbers, and the other two coded for small proteins with less than 25% of the query coverage. This gave us a final selection of 49 genes encoding proteins similar to Pl-LAAO, none of them characterized so far (Supplementary Table S1). All of the detected proteins showed a similarity to the amino oxidase domain pfam01593 in their sequences, and a size between 526 and 685 amino acids (except one protein with 334 amino acids). Interestingly, most of the microorganisms synthetizing these proteins were isolated from seawater samples, which suggests that similar proteins to Pl-LAAO may play a role in the marine environment (Supplementary Table S1).

From all of the detected genomes, *Spirosoma fluviale* DSM 29961 is the only microorganism containing two copies of the genes similar to *Pl-laao*. Regarding their distribution (Table 1), all of them were detected in bacterial genomes, which belonged to three distinct phyla i.e., *Bacteroidetes* (with 18 genes), *Nitrospinae* (1 gene), and *Proteobacteria* (30 genes). Within the latter, 28 genes belonged the *Pseudoalteromonas* genus, which represents the 20.4% of the total sequenced genomes of this taxon. If we consider only the *Pseudoalteromonas luteoviolacea* sequenced genomes, 66.6% of them contained *Pl-laao* similar genes (14/21).

**Figure 7:**
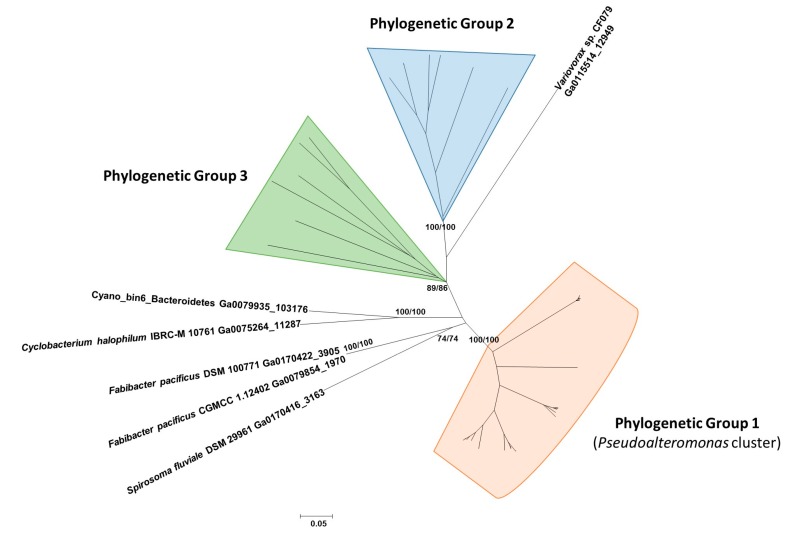
Phylogenetic relationships of Pl-LAAO similar proteins. The tree was created by the neighbor-joining (NJ) method integrated in the program MEGA6. The sequences were aligned using the program MUSCLE built in MEGA6. The evolutionary distances were computed using the p-distance method and are in the units of the number of amino acid differences per site. Numbers at the branches indicate bootstrap values higher than 70% for both NJ and maximum likelihood (ML) trees.

With the aim of investigating the phylogenetic relationships between the proteins similar to Pl-LAAO, they were aligned with MUSCLE (MUltiple SEquence Comparison by Log-Expectation), and then an evolutionary analysis was conducted using the software MEGA6 [28]. Phylogenetic relationships were inferred using both the neighbor-joining (NJ) and maximum likelihood (ML) methods. Three different phylogenetic groups, meeting the criterion of being supported by bootstrap values higher than 70% in both methods, were established (Figure 7).

**Figure 8:**
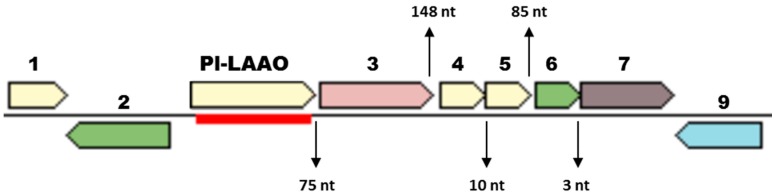
General genome region surrounding *Pl-laao*-like genes in *P. luteoviolacea* strains. (1) Hypothetical protein. (2) Tetratricopeptide repeat (TPR)-like response regulator. (3) Indolepyruvate decarboxylase. (4) and (5) Spondin_N similar proteins. (6) OmpR family response regulator. (7) Signal transduction histidine kinase. (8) Alcohol dehydrogenase. (9) Multidrug and toxic compounds extrusion (MATE) family efflux protein. Color code is for cluster of orthologous groups (COG) function category: Pink—carbohydrate transport and metabolism; green—transcription; gray—signal transduction mechanisms; light brown—unknown.

Group 1 contains 28 proteins, all of them codified by genes from the *Pseudoalteromonas* genus. The proteins synthesized by the *P. luteoviolacea* strains, among them Pl-LAAO, form a well-defined cluster (Supplementary Figure S2). Interestingly, the analysis of the genome region surrounding the genes coding for these *P. luteoviolacea* proteins revealed that all of them showed a similar gene organization (Figure 8). This organization may suggest that *Pl-laao*-like genes could form part of an operon, as they are located together with five other genes similarly orientated with short intergenic regions between each other. One of the genes in the putative operon encodes an indolepyruvate decarboxylase related with the Tryptophan metabolism, two encode proteins with a sequence similarity to Spondin_N pfam06468, and the other two genes code for a response regulator and a histidine kinase, respectively (Figure 8).

Phylogenetic Group 2 comprises eight proteins encoded by bacteroidetes from the *Cytophagales* order, of which six of them are synthesized by bacteria of the genus *Algoriphagus* (Figure S3). All but one presented the conserved COG1231 described in the monoamine oxidases, which is related to amino acid transport and metabolism. Group 3 represents the most heterogeneous group, consisting of seven proteins that belong to three different phyla, namely: *Bacteroidetes*, *Proteobacteria*, and *Nitrospinae* (Figure S4). This may suggest that this group has an ancient evolutionary origin.

**Figure 9:**
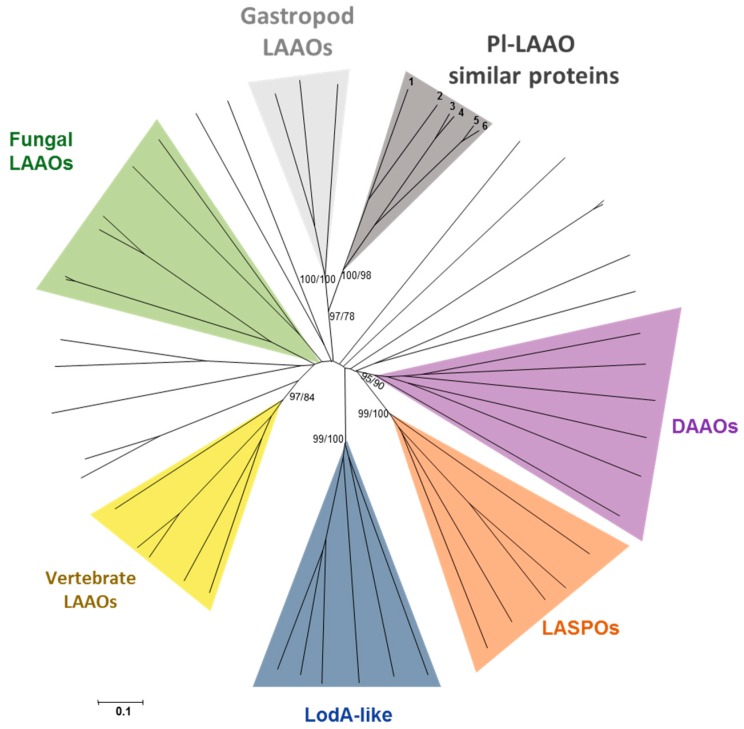
Phylogenetic relationships between proteins similar to Pl-LAAO and representative amino acid oxidases [5]. The tree was created by the NJ method integrated in the program MEGA6. The sequences were aligned using the program MUSCLE built in MEGA6. The evolutionary distances were computed using the p-distance method and are in the units of the number of amino acid differences per site. The numbers at the branches indicate bootstrap values higher than 70% for both of the NJ and ML trees. Among the Pl-LAAO similar proteins, we selected two proteins representing each phylogenetic group. For Group 1: *Algoriphagus* sp. ARW1R1 Ga0206402_101228 (1) and *Spirosoma fluviale* DSM 29961 Ga0170416_5792 (2). For Group 2: *Tenacibaculum ovolyticum* DSM 18103 H518DRAFT_00204 (3) and *Lewinella persica* DSM 23188 B036DRAFT_03117 (4). For Group 3: *Pseudoalteromonas luteoviolacea* CPMOR-2 Pl-LAAO (5) and *Pseudoalteromonas luteoviolacea* 2ta16 PL2TA16_02145 (6). LAAOs—L-amino acid oxidases; DAAO—D-amino acid oxidases; LASPOs—L-aspartate oxidases.

With the intention of shedding light on the evolution of Pl-LAAO similar proteins, we performed a phylogenetic analysis between representative proteins from the three clusters presented above and the characteristic amino acid oxidases previously reported [5]. Among them, the microbial proteins for which the encoding gene has been cloned, as well as other representative proteins from higher organisms are included. The results revealed that the proteins similar to Pl-LAAO constitute a well-defined cluster and are phylogenetically distant from other LAAOs with antimicrobial activity described in marine bacteria, like those belonging to the LodA-like group (Figure 9). In fact, proteins similar to Pl-LAAO are more phylogenetically related to gastropod enzymes than to any other group. This suggests that Pl-LAAO similar proteins and gastropod LAAOs have a common ancestor, which has evolved to meet the LAAO and antimicrobial activity in both phylogenetic clusters.

## 3. Discussion

Several marine microorganisms have been described with an antimicrobial activity, in which distinct compounds of a different nature are involved. *Pseudoalteromonas luteoviolacea* is a marine gammaproteobacteria that had been reported to synthesize antibacterial proteins [16,29]. Here, we report for the first time the identification, cloning, and heterologous expression of the gene encoding this antimicrobial protein in *P. luteoviolacea* CPMOR-2, which has been named Pl-LAAO. Pl-LAAO is a flavoprotein of 653 amino acids that possesses L-amino acid oxidase activity with a broad substrate range (Figure 4). The antimicrobial effect of Pl-LAAO was inhibited by catalase, and is directly proportional to the hydrogen peroxide production, as larger inhibition halos of growth were found with the preferred substrates (Figure 6). This result suggests that the hydrogen peroxide released in the amino acid oxidation is responsible for the antimicrobial activity of Pl-LAAO, in agreement with previous studies [16]. The accumulation of reactive oxygen species like H_2_O_2_ may trigger different forms of cell damage, including lipid peroxidation and DNA strand breakage, which results in bacterial growth inhibition and cell death. However, it cannot be ruled out that other factors apart from H_2_O_2_ could be involved in the antibacterial action of Pl-LAAO. In this sense, it has been described that certain intermediates may potentiate the antimicrobial effect of some lysine alpha-oxidases [27]. In the case of the LAAO from *Trichoderma harzianum*, this enzyme interacts with bacteria, causing membrane permeability [9].

An interesting feature of Pl-LAAO is that it is still active after SDS-PAGE conditions, which allowed for the identification of the protein as well as an estimation of its molecular conformation as a tetramer (Figure 2 and Figure 5). A plausible explanation might be that Pl-LAAO is electronegative at the pH in phosphate buffer, and according to its theoretical pI value, the protein could not be surrounded by SDS, thus avoiding the protein unfolding. This resistance to SDS and β-mercaptoethanol has been described in other flavoproteins with LAAO activity, like the one synthesized by the marine flavobacterium *Aquimarina* sp. antisso-27 [17], or by the fungus *Rhizoctonia solani*, which is indeed activated by SDS [26].

Previously, various microbial LAAOs with a quinone cofactor had been found with antimicrobial activity, such as the L-lysine ε-oxidases from the marine gammaproteobacteria *Marinomonas mediterranea* [30], *Pseudoalteromonas tunicata* D2 [12], *Pseudoalteromonas flavipulchra* JG1 [13] and *Rheinheimera aquatica* GR5 [31]. To the best of our knowledge, this is first report on a FAD-containing LAAO with antimicrobial activity from a marine microorganism. Additionally, no protein with a sequence similarity to Pl-LAAO has been cloned or characterized at the molecular level so far. Hence, a BLASTp was performed against the Integrated Microbial Genomes (IMG) database to detect proteins similar to Pl-LAAO. A total of 49 genes were detected and, according to their phylogenetic distribution, the *Pl-laao* similar genes seem to have no ancient origin as they are not widespread in bacterial genomes, being detected only in three distinct phyla (Table 1). From the 49 detected proteins similar to Pl-LAAO, 28 were encoded by genes detected in the genomes from the genus *Pseudoalteromonas* and, specifically, 14 from *Pseudoalteromonas luteoviolacea* (Table 1). This represents 20.4% and 66.6% of the sequenced strains for each taxon, respectively. The phylogenetic analysis showed that similar proteins to Pl-LAAO from the genus *Pseudoalteromonas* constitute a well-defined cluster of proteins, suggesting that they have a common evolutionary origin (Figure 7 and Supplementary Figure S2). This fact and their high abundance in the *Pseudoalteromonas* genus indicate that this type of proteins may play an important role in these microorganisms, so they have been subjected to a selective pressure to be conserved throughout the evolution, particularly in *P. luteoviolacea*.

Recently, it has been reported that *P. luteoviolacea* CPMOR-2 synthetizes a LAAO very specific for Gly, called PlGoxA [18]. This glycine oxidase contains a cysteine tryptophylquinone (CTQ)-cofactor and belongs to the LodA-like family of quinoproteins [4]. Pl-LAAO and PlGoxA show a low sequence identity (15.4%) and, curiously, Gly was one of the few substrates not oxidized by Pl-LAAO (Figure 4). This indicates that both enzymes may perform complementary roles in CPMOR-2. LodA-like proteins show a L-amino acid oxidase activity, and the hydrogen peroxide produced in the reaction confers to them the antimicrobial properties related to microbial biofilm development and dispersal [12]. Similarly to the *Pl-laao* genes, the occurrence of *lodA-like* genes in sequenced *Pseudoalteromonas* and *P. luteoviolacea* genomes is also very high (36.5% and 90.5%, respectively). In fact, all of the detected *Pseudoalteromonas* genomes containing *Pl-laao* genes also presented one or various *lodA-like* genes. Further studies are necessary in order to understand the relationship between these proteins, which show important biochemical differences in terms of the cofactor used, but have evolved analogously to have LAAO and antimicrobial activities.

Regarding the physiological function of Pl-LAAO, several possibilities can be considered based in the enzymatic reaction catalyzed. Several fungal LAAOs have been proposed to be involved in the utilization of amino acids as a nitrogen source. Similarly to Pl-LAAO, they have a broad substrate range allowing the use of several amino acids for growth [32,33]. In addition, the presence of a gene encoding a putative protein similar to an indolepyruvate decarboxylase related to Trp metabolism, in the same operon as *Pl-laao* (Figure 8), suggests the involvement of Pl-LAAO in such a catabolic role as after the amino acid deamination performed by the LAAO, a keto acid would be generated. On the other hand, the generation of hydrogen peroxide and the microbial activity associated suggest that Pl-LAAO and similar proteins could take part in the microbial interaction and competition in microbial communities, and therefore they may play an ecological role in such interactions. In this regard, the LAAOs from different gastropods, like the sea hare *Aplysia californica* and the giant snail *Achatina fulica*, have been found to provide protection against invading bacteria [24,34]. In fact, the phylogenetic analysis revealed that Pl-LAAO and alike proteins are more phylogenetically related to gastropod LAAOs than to any other kind of enzymes described with amino acid oxidase activity (Figure 9). This suggests that Pl-LAAO similar proteins and gastropod LAAOs might have a common evolutionary origin, raising interesting questions about the physiological relevance of these enzymes. Interestingly, the *Pl-laao* genes were found not only in the genomes from the *Pseudoalteromonas* species, but also in other microorganisms related to host-associated microbial communities from marine organisms, such as algae, plants, cnidarian, or fishes (Supplementary Table S1). LAAOs with a quinone cofactor from the marine gammaproteobacteria *P. tunicata* and *M. mediterranea* [12] play a role in the development of microbial biofilms in those surfaces, so it cannot be ruled out that they play a similar role to the proteins described in this study.

In conclusion, this work shows the identification, cloning, partial characterization, and evolutionary analysis of Pl-LAAO, the antimicrobial protein described in *P. luteoviolacea* CPMOR-2. We believe that our findings provide insights into the biological and ecological significance of antimicrobial LAAOs in the *Pseudoalteromonas* genus. The approach and methods described in this investigation may be used as a guide for the detection of new antimicrobial LAAOs, whose study may be useful for biotechnological applications.

## 4. Materials and Methods

### 4.1. Strains, Culture Media, Plasmids, and Primers

The bacterial strains, plasmids, and primers used in this study are listed in Table 2. *Pseudoalteromonas luteoviolacea* CPMOR-2 was isolated from seawater samples [16]. This strain was usually incubated in a liquid marine media at 25 °C and 130 rpm. Different media were used, as follows: Marine broth 2216 (Difco), complex Marine Medium (MMC) [35], chemically minimal medium MN [36], MNG (MN plus glucose) [37], and MNGY (MNG plus 0.01% of yeast extract). *Escherichia coli* strains were grown in a Luria–Bertani (LB) medium at 37 °C and 250 rpm. The LB and M9 chemical defined medium [38] were used for the antibiograms. When required, the media were supplemented with the appropriate antibiotic (Sigma-Aldrich, St. Louis, MO, USA).

### 4.2. DNA Manipulations

The DNA was manipulated according to standard protocols [38]. The *Pl-laao* gene was amplified from the genomic DNA of the *P. luteoviolacea* CPMOR-2 genome, using the primers indicated in Table 2, and the KOD DNA polymerase (Merck, Darmstadt, Germany). The restriction enzymes were purchased from Fermentas (Thermo-Fischer Scientific, Waltham, MA, USA). The DNA restriction fragments were eluted from agarose gels by utilizing Qiaquick columns (Qiagen, Venlo, The Netherlands), and were cloned into a Novagen pET15b plasmid (Merck, Darmstadt, Germany) using the T4 DNA ligase from Invitrogen. The transformation of *E. coli* CD03 was carried out by electroporation [40]. The isolation of plasmid DNA from *E. coli* was achieved using the Wizard Plus SV Minipreps DNA Purification System from Promega. The construction was checked by sequencing.

### 4.3. Expression and Purification of Recombinant Protein

One colony of *Escherichia coli* strain CD03 containing pET15b with *Pl-laao* gene fused to a N-terminal hexahistidine tag was incubated until it reached an OD_600_~0.6 in an LB medium, with the addition of 50 μg/mL ampicillin at 37 °C and 250 rpm. Next, the protein expression was induced with isopropyl-β-d-thiogalactopyranoside (IPTG) 1 mM. Then, the cultures were incubated overnight at 25 °C, before they were harvested by centrifugation at 5000× *g* for 10 min. The cell pellet was resuspended in a binding buffer (50 mM sodium phosphate, 500 mM NaCl, 20 mM imidazole, pH 7.4) and was disrupted by sonication using a Braun Labsonic M sonicator. The homogenate was centrifuged at 13,000× *g* for 2 min, and the pellet was discarded. A cell lysate containing the soluble proteins was applied to columns with 1.5 mL of Qiagen Ni-NTA resin. The proteins attached to the column were eluted with an elution buffer (50 mM sodium phosphate, 500 mM NaCl and 500 mM imidazole, pH 7.4). Amicon^®^ Ultra centrifugal filters 30K (Merck, Darmstadt, Germany) were applied to remove the imidazole.

### 4.4. SDS-PAGE

Sodium dodecyl sulfate polyacrylamide (SDS-PAGE) was performed by the method of Laemmli (1970) [41]. The stacking and separating gels consisted in acrylamide of 3% and 8%, respectively. The running buffer was 0.3% Tris, 1.44% glycine, and 0.1% SDS, with pH 8.3. The samples were mixed with a 1/2 volume of loading buffer containing 3 M 2-mercaptoethanol, 0.18 M Tris-HCl, pH 6.8, 15% glycerol, 0.075% bromophenol blue, and 9% SDS. The gels were run at a low voltage (70–90 V). After electrophoresis, the gels were stained with Coomassie brilliant blue R250. Duplicate lanes with identical samples were fixed for 2 h in a solution of 10% acetic acid and 20% isopropanol, and were washed for 2 h in deionized water in order to detect the antibacterial activity of the bands [42]. These conditions utilized, without boiling the samples with the proteins, which were previously reported as non-denaturing conditions for the detection of antimicrobial and amino acid oxidase activity [19], were non-denaturing conditions for assessing the Pl-LAAO activity as well. The boiling of the sample resulted in the denaturalization of the protein.

### 4.5. Activity Assays

To detect the amino acid oxidase activity, a fluorimetric assay for the determination of the H_2_O_2_ production was routinely used (Amplex Red hydrogen peroxide/peroxide assay; Invitrogen) [6]. The assay mixture (100 μL) contained the substrate (at the specified concentration) in a 50 mM sodium phosphate buffer with NaCl 0.5 M pH 7.4, 0.05 mM Amplex Red, 0.1 U/mL of peroxidase, and 10 μL of sample. Reactions were carried out at 37 °C for 15 min in 96-well microplates. Amplex Red oxidation was followed using an excitation filter of 550 nm and an emission filter of 590 nm in a FLUOstar Optima (BMG LabTech, Ortenborg, Germany). The activities were normalized according to the milligrams of protein present in each sample, and were measured by the Bradford assay (Sigma, St. Louis, MO, USA). The fluorometric assay was also used to determine directly the amino acid oxidase activity in the fragments sliced from SDS-PAGE, which were previously fixed and washed with deionized water, as previously described [43].

The antibacterial activity was assayed through antibiograms. A suspension of *E. coli* UM202 [39] in NaCl 0.85% (OD_600_ = 0.21) was seeded on LB and M9 plates. In some experiments, 20 μL of purified protein, catalase or substrate were loaded into 6 mm disks of Filter Paper Backing (BioRad, Hercules, CA, USA) and allowed to air dry, before placing them onto the agar plate. To determinate the antimicrobial activity of the proteins run by SDS-PAGE, the gel was sliced and placed onto the antibiogram plate after fixing and washing [43]. The antibiograms plates were incubated for 48 h at 25 °C.

### 4.6. UV-VIS Spectrum

The experiments were performed with purified Pl-LAAO in 50 mM phosphate buffer (pH 7.4) The emission spectra were recorded in a quartz cuvette with a path length of 10 mm using a FLUOstar OPTIMA fluorescence spectrometer (BMG Labtech, Ortenborg, Germany) at 25 °C, and were then subjected to ultraviolet-visible absorption spectrum analysis. A mixture without Pl-LAAO was used as a negative control.

### 4.7. Mass Spectrometry Analysis

The mass spectrometry analysis of protein gel fragments, which consisted of in-gel trypsin digestion followed by a HPLC-MS/MS analysis, was performed as previously described [19].

### 4.8. Detection, Alignment, and Phylogenetic Analysis of Pl-LAAO Similar Proteins

In order to identify and analyze the proteins similar to Pl-LAAO (accession KZN49687) in the microbial genomes, the tools available at the Integrated Microbial Genomes Expert Review (IMG/MER) were used [44]. BLASTp search at IMG/MER, using an E-value cut of 1e^−10^ and a minimum identity percentage of 30%, was performed with the Pl-LAAO sequence as a query (accession KZN49687). Peptide sequences similar to Pl-LAAO were aligned using MUSCLE (MUltiple Sequence Comparison by Log-Expectation) [45] and were then incorporated into the program MEGA6 [28] in order to perform the phylogenetic analysis. The phylogenetic relationships were computed using both the neighbor-joining (NJ) and maximum likelihood (ML) methods. The distances between the sequences were computed using the p-distance method and are in the units of the number of amino acid differences per site. The reliability of each node in the tree constructed was estimated using a bootstrap analysis with 500 replicates. The proteins analyzed in this study were clustered in different phylogenetic groups, which met the criterion of being supported by a bootstrap analysis with a higher than 70% reliability in both of the NJ and ML trees.

## Figures and Tables

**Table 1:** Occurrence of genes similar to *Pl-laao* in microbial genomes deposited in the Integrated Microbial Genomes (IMG) database as of 14 September 2018, according to their phylogenetic distribution. Phyla are in bold and classes marked with an asterisk. The number between brackets indicates the total number of sequenced genomes for each taxon at the moment of the analysis.

Taxon	Phylogenetic Group 1	Phylogenetic Group 2	Phylogenetic Group 3	Ungrouped
***Proteobacteria* (29907)**	**28**	**0**	**1**	**1**
* *Alphaproteobacteria* (4218)	0	0	1	0
* *Gammaproteobacteria* (19977)	28	0	0	0
* *Betaproteobacteria* (3182)	0	0	0	1
***Bacteroidetes* (2483)**	**0**	**8**	**5**	**5**
* *Saprospiria* (15)	0	0	2	0
* *Flavobacteriia* (1009)	0	0	3	0
* *Cytophagia* (229)	0	8	0	4
* Unclassified (216)	0	0	0	1
***Nitrospinae* (54)**	**0**	**0**	**1**	**0**
* *Nitrospinia* (21)	0	0	1	0
**TOTAL**	**28**	**8**	**7**	**6**

**Table 2:** Bacterial strains, plasmids and primers used in this work.

Strains	Relevant Genotype and Description, or Sequence	Reference or Source
*Pseudoalteromonas luteoviolacea* CPMOR-2	Wild-type	[16]
*Escherichia coli* CD03	BL21(DE3) katE12::Tn10 katG::Tn5, [Cat^+/−^]	[25]
*Escherichia coli* UM202	MP180 *katG*::Tn*10*, [Cat^+/−^]	[39]
**Plasmids**		
pET15b	pET15b	Novagen
pETpl-laao.15	pET15b, *Pl-laao*	This study
**Primers ^1^**		
AminoORCPMOR2Nde (D)	5′-AAGGAATACATATGACACATTATACTTTTGG-3′	
AminoORCPMOR2Bam (R)	5′-CTTCTAACGGATCCTTAAAGTAATCTG-3′	

^1^ D: direct primer; R: reverse primer; Restriction sites are underlined; [Cat^+/−^]: decreased catalase activity.

## Supplementary Materials

The following are available online at http://www.mdpi.com/1660-3397/16/12/499/s1: Figure S1. LAAO activity in the supernatants of strain CPMOR-2 cultivated in different media at the stationary phase. The LAAO activity was assayed using casamino acids of 2% as a substrate. Figure S2. Phylogenetic relationships of Group 1, proteins similar to Pl-LAAO in the genus *Pseudoalteromonas*. The tree was created by the NJ method integrated in the program MEGA6. Sequences were aligned using the program MUSCLE built in MEGA6. The evolutionary distances were computed using the p-distance method and are in the units of the number of amino acid differences per site. Numbers at the branches indicate bootstrap values higher than 70% for both NJ and ML trees. Pl-LAAO is indicated in red. Figure S3. Phylogenetic relationships of Group 2 of proteins similar to Pl-LAAO. The tree was created by the NJ method integrated in the program MEGA6. Sequences were aligned using the program MUSCLE built in MEGA6. The evolutionary distances were computed using the p-distance method and are in the units of the number of amino acid differences per site. Numbers at branches indicate bootstrap values higher than 70% for both the NJ and ML trees. Figure S4. Phylogenetic relationships of Group 3 of proteins similar to Pl-LAAO. The tree was created by the NJ method integrated in the program MEGA6. Sequences were aligned using the program MUSCLE built in MEGA6. The evolutionary distances were computed using the p-distance method and are in the units of the number of amino acid differences per site. Numbers at branches indicate bootstrap values higher than 70% for both NJ and ML trees. An asterisk indicates that this branch was not detected, or it had a value lower than 70% in one of trees. Table S1. Similar proteins to Pl-LAAO, deposited in the Integrated Microbial Genomes (IMG) database as of 14 September 2018.
