# Supplementary material for: The Pseudoalteromonas luteoviolacea L-amino Acid Oxidase with Antimicrobial Activity Is a Flavoenzyme
Source: Mar Drugs. 2018 Dec 12;16(12):499. doi: 10.3390/md16120499 (PMC6316408; doi:10.3390/md16120499)
Supplement: Supplementary file 1 [file marinedrugs-16-00499-s001.zip › marinedrugs-398624-supplementary.pdf]

Supplementary Materials

The *Pseudoalteromonas luteoviolacea* L-amino Acid Oxidase with Antimicrobial Activity is A Flavoenzyme

Andrés Andreo-Vidal [1], Antonio Sanchez-Amat [1,*] and Jonatan C. Campillo-Brocal [1,2,*]

[1] Department of Genetics and Microbiology, University of Murcia, Murcia 30100, Spain; andres.andreo@um.es (A.A.-V.)

[2] Department of Industrial Biotechnology, School of Engineering Sciences in Chemistry, Biotechnology and Health (CBH), KTH Royal Institute of Technology, AlbaNova University Center, SE-106 91, Stockholm, Sweden

* Correspondence: antonio@um.es (A.S.-A.); jonatancristian.campillo@um.es (J.C.C.-B.); Tel. +34-868-884-955 (A.S.-A.); +34-606-634-475 (J.C.C.-B.)

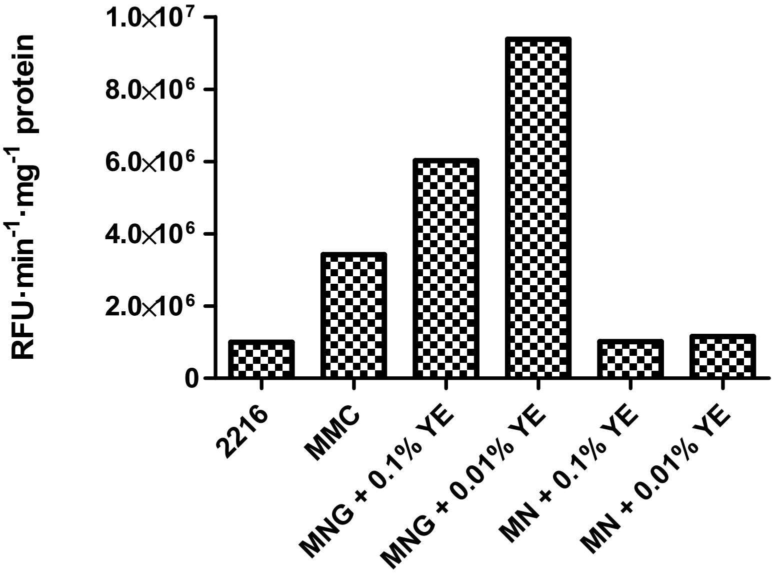

Figure S1. LAAO activity in the supernatants of strain CPMOR-2 cultivated in different media at the stationary phase. LAAO activity was assayed using casamino acids 2% as a substrate.

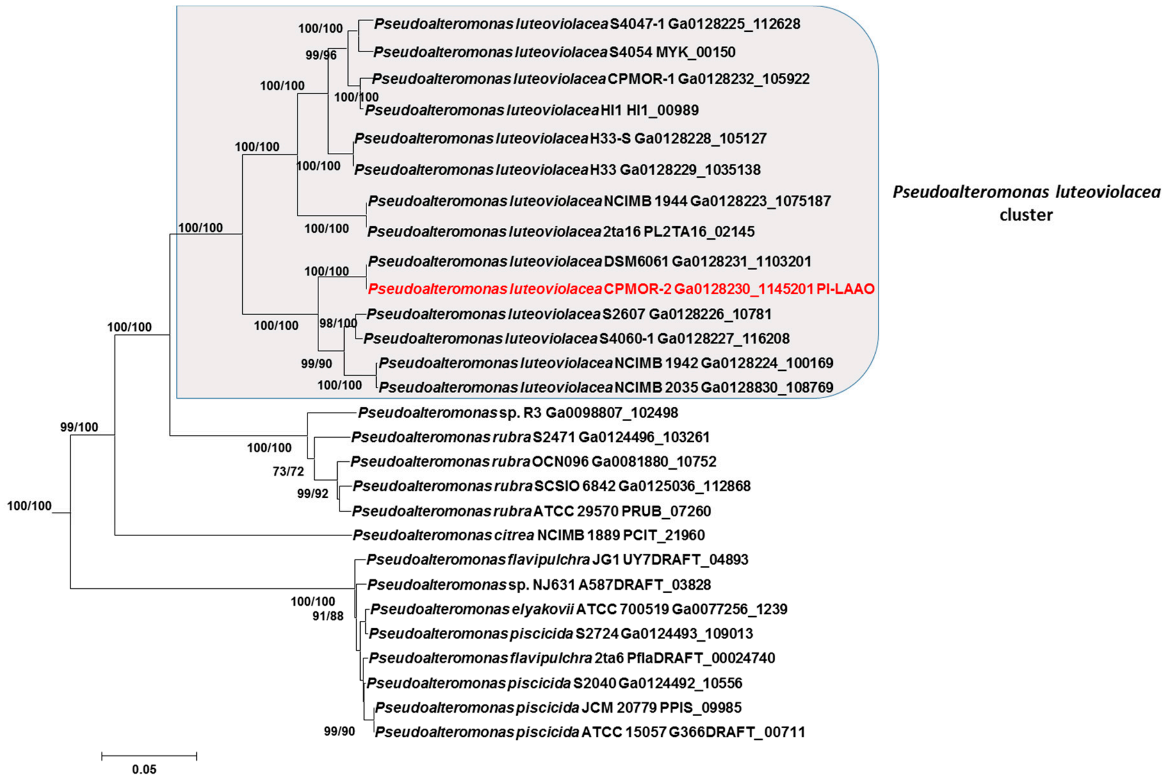

Figure S2. Phylogenetic relationships of Group 1, proteins similar to Pl-LAAO in the genus *Pseudoalteromonas*. The tree was created by the NJ method integrated in the program MEGA6. Sequences were aligned using the program MUSCLE built in MEGA6. The evolutionary distances were computed using the p-distance method and are in the units of the number of amino acid differences per site. Numbers at branches indicate bootstrap values higher than 70% for both NJ and ML trees. Pl-LAAO is indicated in red.

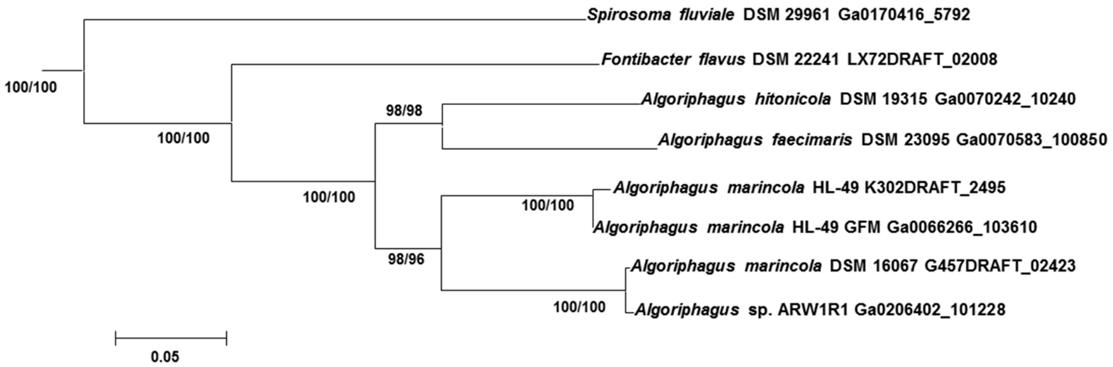

Figure S3. Phylogenetic relationships of Group 2 of proteins similar to Pl-LAAO. The tree was created by the NJ method integrated in the program MEGA6. Sequences were aligned using the program MUSCLE built in MEGA6. The evolutionary distances were computed using the p-distance method and are in the units of the number of amino acid differences per site. Numbers at branches indicate bootstrap values higher than 70% for both NJ and ML trees.

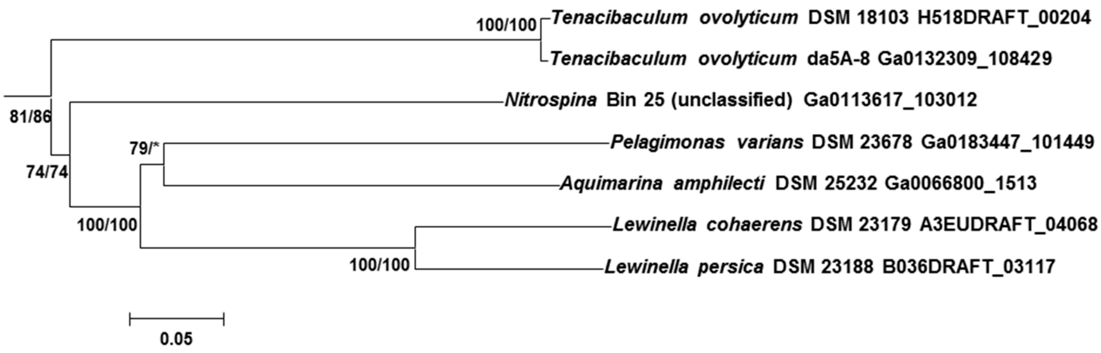

Figure S4. Phylogenetic relationships of Group 3 of proteins similar to Pl-LAAO. The tree was created by the NJ method integrated in the program MEGA6. Sequences were aligned using the program MUSCLE built in MEGA6. The evolutionary distances were computed using the p-distance method and are in the units of the number of amino acid differences per site. Numbers at branches indicate bootstrap values higher than 70% for both NJ and ML trees. An asterisk indicates that this branch was not detected, or it had a value lower than 70% in one of trees.
